# Changing bodies: A scoping review and thematic analysis of family experience during serious childhood illness

**DOI:** 10.1177/13674935231168683

**Published:** 2023-04-07

**Authors:** Emma Maynard, Megan Bennett

**Affiliations:** 1 Dept of Child & Family Health & Methodologies Division, 4616 King’s College London, London, UK; 2 School of Education & Sociology, 171171 University of Portsmouth Faculty of Humanities and Social Science, Portsmouth, UK

**Keywords:** Parents, nursing, childhood illness, family

## Abstract

This scoping review has investigated experiences of children and parents encountering in-patient treatment for serious childhood illness, including current or potential use of technology as a support mechanism. The research questions were 1. What do children experience during illness and treatment? 2. What do parents experience when their child is seriously ill in hospital? 3. What tech and non-tech interventions support children’s experience of in-patient care? The research team identified *n* = 22 relevant studies for review through JSTOR, Web of Science, SCOPUS and Science Direct. A thematic analysis of reviewed studies identified three key themes reflecting our research questions: *Children in hospital, Parents and their children,* and *Information and technology*. Our findings reflect that information giving, kindness and play are central in hospital experiences. Parent and child needs in hospital are interwoven and under researched. Children reveal themselves as active producers of pseudo-safe spaces who continue to prioritise normal child and adolescent experiences during in-patient care.

## Introduction

This review was subcontracted to the research team by Coggi Ltd, who are a health tech start-up, funded by Innovate UK. Our objective was to synthesise existing evidence about needs and experiences of children aged 5 to 12 and their parents during in-patient treatment for serious childhood illness. This evidence is intended for use in development of a web-based Application (App) called Coggi which will support children receiving treatment by offering age-appropriate information and online game-play opportunities.

Numbers of children experiencing serious illness, including those which are life limiting and life threatening, continue to rise annually (Heath et al., 2022). Despite increasing survival rates, seriously ill children experience a multitude of restrictions through pain, fatigue and time lost from school and social activities (Heath et al., 2022). They encounter disruption to their social and emotional development and learning (Kirk et al., 2019; Jasem et al., 2022), and are further impacted by social pressures which position difference as inherently wrong (Passos dos Santos et al., 2022; Heath et al., 2022).

Overall, childhood illness is associated with stress, pain, confusion and fear both for children and their families (Lindström Nilsson et al., 2020; Wangmo et al., 2016; Price et al., 2022). Past scoping reviews have considered experiences of children in hospital in terms of play and arts-based interventions in hospital. These reviews reinforce the importance of play and distraction from medical intervention (Gjærde et al., 2021; Fancourt and Finn, 2019), however they lack synthesis with other aspects of support. Coad and Shaw (2008) and Davison et al. (2021) have conducted scoping reviews into children’s voice and child-centred care, concluding that there is a greater rhetoric about this than actual involvement of children in care planning and delivery. Functional and physical tasks of healthcare settings continue to take precedence, and although child-centred practice is often intended, it is not always delivered upon (Coad and Shaw, 2008; Davison et al., 2021). Scoping reviews by Maalouf et al. (2018) and Dawe et al. (2018) focused on in-hospital use of social robots and identified these as a source of comfort for children, especially when parents were absent. Pelentsov et al.’s (2015) scoping review identified a dearth of attention towards parents’ own needs and proposed a framework for support to include informational, emotional, social, practical, physical and psychological needs.

Overall, the literature reflects a partial representation of family needs during serious childhood illness. A focus on combined experiences of children and parents is lacking, and so is research about specific health-related uses of technology in addressing some areas of need, that is, information giving and play (Dawe et al., 2018). Our research questions were developed from our literature review and were confirmed as: 1. What do children experience during illness and treatment? 2. What do parents experience when their child is seriously ill in hospital? 3. What tech and non-tech interventions support children’s experience of in-patient care?

### Aim

To identify needs and experiences of children and parents during in-patient treatment for serious childhood illness, to inform development of the Coggi App.

## Method

**Figure 1.**
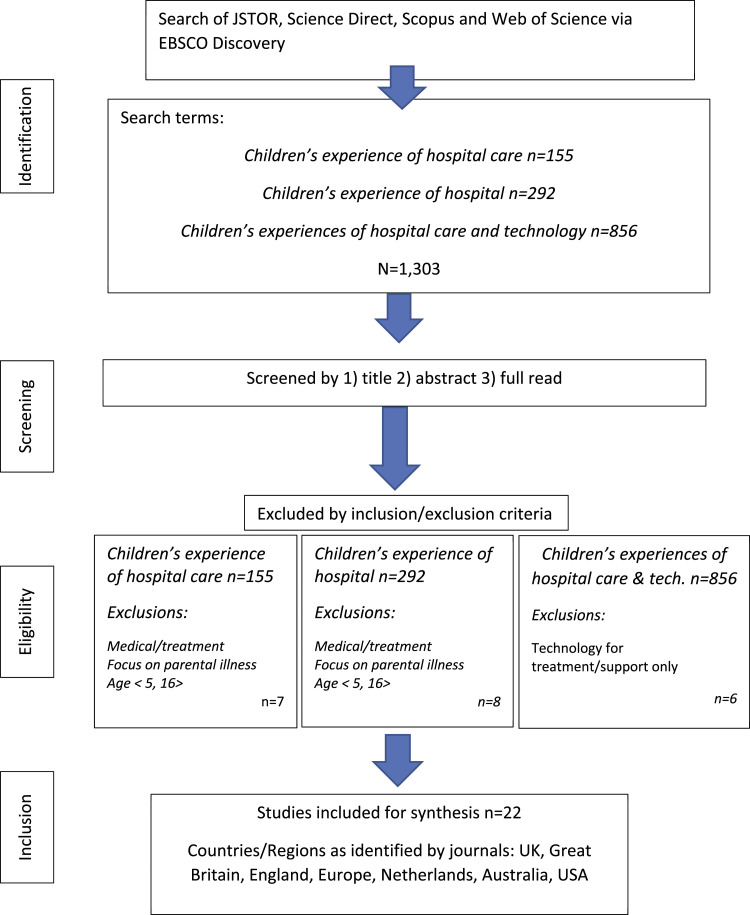
Flowchart charting search and inclusion of papers by inclusion criteria.

We used Levac et al.’s (2010) extension of Askey and O’Malley’s (2005) recommendations for scoping studies. The first author (Emma Maynard) collaborated with Coggi Ltd to build relationships, share ideas and determine our focus. We aimed to provide Coggi Ltd with a synthesised review of current research about children’s in-patient experience, to develop a first stage design of the App. Inclusion and exclusion criteria were agreed in collaboration with Coggi Ltd Supplementary files Figure 1: *Flowchart charting selection of papers for review by inclusion criteria*, and Table 1: *Use of* Levac et al.’s (2010) *Recommendations for Scoping Reviews* explain the process by which the review was conducted resulting in a sample of *n* = 22 studies. In summary, the review process entailed 1. Defining research questions; 2. Identifying relevant articles; 3. Study selection; 4. Charting data; 5. Collating, summarising and reporting results; 6. Consulting with stakeholders (Levac et al., 2010; Davison et al., 2020). Researchers met to review studies by title and abstract, and re-confirmed criteria to identify studies for inclusion and synthesis through full read-throughs. The team met regularly and compared observations through annotations (Levac et al., 2010).

**Table 1.** Use of Levac et al.’s (2010) recommendations for scoping reviews.

Framework stage (recommendations of Levac et al. (2010) in summary)	Changing bodies scoping Review steps: (Authors)
#1 Identifying the research question to consider concept, population, and specific focus. Consider purpose and intended outcomes, and rationale	The research questions were discussed with our funders, Coggi Ltd, to determine the specific research questions including the age range of children considered within studies. The intended outcome was to enable Coggi Ltd to access research findings and incorporate this into the development of their application to ensure their provision for children was research informed
#2 Identifying relevant studies, guided by research question with a suitable team. Ensure any limitations can be justified	Following #1, the research team established inclusion and exclusion criteria and began the search
#3 Study selection through an iterative process, searching, refining and reviewing for inclusion. The team should meet and discuss inclusion and exclusion criteria. Abstracts should be reviewed independently by researchers, and then full articles. Researchers should meet regularly during the process, a third reviewer could be called upon to resolve disagreement	Both members of the research team were involved throughout. We discussed the possibility of disagreement and agreed we would ask for a colleague’s opinion if needed, however, given the fairly straightforward inclusion criteria we felt this was unlikely and no such issues were encountered. Both researchers reviewed each abstract. Those identified for full reads were also considered by both researchers, prior for inclusion for synthesis (see Figure 1: Flowchart)
#4 Charting the data; an iterative process to decide which aspects of data to extract in order to address the research question, and chart emergent data qualitatively to ensure the approach is likely to address research questions	Both researchers made notes on each paper and met at regular intervals to discuss observations, ensuring the selected papers were in line with the research questions and consultation with the funder
#5 Collating summarizing and reporting results; 3 stages of analysis through numerical summary and qualitative thematic analysis; reporting the results to refer to the research question; consider the meaning of findings in relation to the research question	Numerical summaries were not suitable or helpful for our analysis. We undertook qualitative thematic analysis. Themes were identified which addressed the research questions as a whole, which enabled us to capture the nuances of the studies rather than attempting a more restrictive reporting of qualitative data by individual question
#6 Consultation with stakeholders. Preliminary findings to inform ongoing consultation. Clearly articulate process to stakeholders and incorporate knowledge transfer with stakeholders	Preliminary findings were presented to stakeholders. This enabled us to decide collectively that findings were relevant to the intended outcomes, and addressed the research questions. Additionally we were able to acknowledge any unanticipated findings

Studies focused on children aged 5 to 12 were most suitable to Coggi Ltd’s needs given age parameters set by them with their funder, Innovate UK. Some studies we identified focused on children aged 5 to 12 *and* children older than 12. After discussion with Coggi Ltd it was decided that these were relevant and should be included. We limited the search to 10 years since publication (2012) to capture a contemporary picture of in-patient experiences with the potential to reflect younger children’s use of smartphone technology (Terras and Ramsay, 2016).

Relevant papers were analysed for review through the University EBSCO database, which trawled JSTOR, Web of Science, Scopus and Science Direct to answer our research questions. Initially we intended to scrutinise differences pertaining to gender, ethnicity and diagnosis; however, we were not able to identify studies which considered this and therefore disregarded this question.

### Inclusion criteria

Peer reviewed research conducted into lived experiences of childhood illness, diagnosis and treatment over the last 10 years, and written in English which centralises child and parent experience, considers the possible impact of childhood illness, diagnosis and treatment, and/or, researches use of technology to support children’s mental health and wellbeing. Exclusion criteria discounted research which exceed 10 years since publication, or which does not centralise child and parent experiences, or was not published in English.

### Analysis

Thematic analysis was used to synthesise the studies following Braun and Clarke’s (2006) 6-Step process; 1. Familiarisation with the data, 2. Generating initial codes, 3. Searching for themes, 4. Reviewing themes, 5. Defining and naming themes and 6. Producing the report. Steps 1–5 were carried out by both authors, including mutual checking and cross referencing to our research questions. Preliminary findings were shared with Coggi Ltd to confirm relevance to our intended outcome. Table 2 (supplementary file) presents our Table of Themes following thematic analysis of reviewed studies. Themes at ordinate level were organised into three superordinate themes: *Children in Hospital*, *Parents and their children*, and *Information and Technology*. Our sample reflected both curative and non-curative conditions including cancer, disability, sickle cell disease, stroke, MS, congenital disorders and children undergoing haematopoietic stem cell transplantation (HSCT).

**Table 2.** Table of themes.

Ordinate theme	Relevant papers	Superordinate themes
Children’s emotional lives	Plummer et al. (2021)	Children in hospital
	Kassa et al. (2017)	
	McKevitt et al. (2018)	
	Bruins Slot et al. (2018)	
	Lindstrom et al. (2020)	
	Shilling et al. (2012)	
	Constantinou et al. (2021)	
A changed and changing body	Lewis et al. (2016)	
	Kirk et al. (2019)	
	Witt et al. (2019)	
	Lindstrom et al. (2020)	
	Bryan et al. (2019)	
	McKevitt et al. (2018)	
	Nicholas and Chahauver (2017)	
	Lambert et al. (2014)	
Space, place and play	Crossen et al. (2022)	
	Bryan et al. (2019)	
	Lindstrom et al. (2020)	
	Jasem et al. (2022)	
	Plummer et al. (2021)	
	Lambert et al. (2014)	
	Shilling et al. (2012)	
Parents and their children	Lernevall et al. (2021)	Parents and their children
	Foster et al. (2016)	
	Darling et al. (2019)	
	Jasem et al. (2022)	
	Bruins Slot et al. (2018)	
Information giving and gathering	Wangmo et al. (2016)	Information and technology
	Bryan et al. (2019)	
	Lewis et al. (2016)	
	Foster et al. (2016)	
	Crossen et al. (2022)	
Technology aided support	Huby et al. (2017)	
	Moerman et al. (2021)	
	Nicholas and Chahauver (2017)	
	Moar & Mitchem (2015)	
	Bergin and Davies (2020)	
	Sawesi et al. (2016)	

### Findings

Our findings synthesise studies which report on emotional, treatment and support experiences of children and parents during serious childhood illness. Ways in which information was communicated appeared pivotal in hospital experiences and included interpersonal skills, timeliness, privacy and accessibility. Space to play and feel safe were extremely important however this was sometimes compromised. Our analysis is presented below, with discussion of each ordinate theme nested within each superordinate theme. Only one theme was identified for parents’ needs which reflect limited research in this area, and consistency of those findings.

## Children in hospital

### Children’s emotional lives

Our sample convey strong messages about the impact of serious childhood illness on children’s mental health, social development and learning (Kirk et al., 2019; Bryan et al., 2019; Jasem et al., 2022). Significant psychological problems are evident, including suicidal ideations, self-harm, aggression, changes in personality (McKevitt et al., 2018) and sadness (Lindström Nilsson et al., 2020). Sick children experience anxiety (Bryan et al., 2019), fears about needles and about not waking up after procedures (Kassa et al., 2017). Psychological pain is deemed greater and longer lasting than physical pain; the fear of pain worse than pain itself (Plummer et al., 2021). Despite these distressing experiences, some positive experiences of hospital care are also reported, such as playrooms, supportive staff, happiness, feeling praised for bravery (Shilling et al., 2012) and feeling special (Witt et al., 2019). Children with sickle cell disease and liver transplants appear to accept and adapt to their illness, continuing to live full lives (Constantinou et al., 2021; Wright et al., 2015).

### A changed and changing body

*A changed and changing body* describes seriously ill children, disrupted from normal life, but still developing in broadly normal ways. Kirk and Hinton's (2019) study focuses on children aged 10–19 with multiple sclerosis (MS), noting that their bodies change continuously due to their MS, a feature also identified by McKevitt et al.’s (2018) study about childhood strokes. At key times of child development, especially puberty, bodily changes are prominent in children’s minds (Maynard et al., 2020). McKevitt et al. (2018) and Kirk et al. (2019) echo this, demonstrating duality between normal physical development, and changes incurred through illness. Children’s re/positioning of their changed bodies is evident through their use of online peer support. They overtly lessen stigma while strategizing together about self-presentation and inclusion in mainstream peer contexts (Lewis et al., 2016; Witt et al., 2019). Avoidance is identified as an active point of rebellion in lives heavily controlled through treatment regimes, in order to feel more normal despite visible differences between sick children and their peers (Wright et al., 2015; Kirk et al., 2019).

### Space, place and play

Play is heavily represented in the review with reports of playrooms, medical clowns and animal therapy which enable respite (Bruins Slot et al., 2018; Lernevall et al., 2021; Lindstrom Nillson et al., 2020; Constantinou et al., 2021). Children enjoy medical clowns prior to, but not during treatment, and lower key interactions which stimulate the senses are preferred to boisterousness (Bruins Slot et al., 2018). Fun, distraction and affection were gained from a therapy dog (Lindström Nilsson et al., 2020).

There are clear messages about how children experience hospital treatment spaces. Plummer et al. (2021) examine how children undergoing invasive treatment recreate pseudo-safe spaces by retreating under a blanket or rolling into a ball when a medical practitioner approaches. Shilling et al. (2012) identify overt behaviours as children recreate their own personalised space in hospital, particularly with disabled children who spend extended time in these settings, and for whom camaraderie between hospital peers seems especially evident. Children identify bathrooms as the only space unlikely to be invaded (Plummer et al., 2021; Shilling et al., 2012).

## Parents and their children

Parents’ experiences during their child’s illness are a key part of this review and representations of ‘parent and child’ appear symbiotic. Parents experienced their child’s distress as their own, quoting ‘if s/he gets better, we get better’ (Foster et al., 2016: 116). Parents experience vicarious trauma and acute stress when an illness is life threatening, describing life as before-and-after, demarcating pre and post diagnosis (Lernevall et al., 2021; Darling et al., 2019). Where offered, play interventions such as medical clowns support the parent with brief respite (Lernevall et al., 2021). Foster et al. (2016) notes parents reflect some gaps in care for children’s emotional trauma due to healthcare teams prioritising physical care.

We note evident disparity in treatment of mothers and fathers. Fathers are apparently less likely to accompany children to hospital during a crisis, due to mothers being prioritised in ambulances with limited space. This priority continues throughout admission, assessment and ongoing communication, especially when parents were divorced (Lernevall et al., 2021; Foster et al., 2016). This disparity results in delayed and longer lasting depressive symptoms for fathers (Darling et al., 2019).

## Information and technology

### Information giving and gathering

Appropriate and carefully communicated information is essential for both children and parents. Consistent information for parents is paramount in enabling trust in medical teams. Inconsistent information leads directly to mistrust, and a suspicion of a poor prognosis (Foster et al., 2016; Darling et al., 2019). Wangmo et al. (2016) note additional strain on parents waiting for translators. Parents and children associate information with a sense of control. Such control enables parents to provide their child with calm support, especially where the situation is dramatically unfamiliar. In this case, specific types of information are needed; what to expect on the ward as well as specific information about their child (Lernevall et al., 2021). Sudden unexpected changes in treatment or discharge plans are met with alarm and confusion for parents and children (Wangmo et al., 2016; Lernevall et al., 2021).

Children are represented as potential care partners with a need for full, clear information (Huby et al., 2017; Wangmo et al., 2016). Although parents advocate children’s involvement in decision making, they simultaneously seek to avoid burdening them with information as it can also provoke anxiety (Huby et al., 2017; Shilling et al., 2012; Bryan et al., 2019). Open communication involving children relies heavily on the engagement of medical staff and not all were found to be approachable (Crossen, 2022). Bryan et al. (2019) conjecture that even with approachable staff, children are reticent about speaking openly in ward environments due to risks of being overheard.

While some studies reflect dynamic and creative communication (Stewart et al., 2013; Huby et al., 2017), Bryan et al. (2019) found variability in nurse-child communication: Nurses carefully explain and seek children’s consent for recording body weight, but complete other checks without forewarning. Overall, information sharing practices appear pivotal in confirming hospital experiences as either positive or negative. Positive experiences are associated with friendly, humorous and caring staff. Negative associations regard *controlling* staff with *poor communication*, leading to frightening experiences (Plummer et al., 2021; Shilling et al., 2012).

### Technology aided support

Proactive information gathering is evident in children’s use of technology. Huby et al. (2017) notes that as digital natives, children are likely to consult the Internet about their conditions. This is risky, however, as web content may not be age appropriate, reliable, or condition specific (Stewart et al., 2013). Children select applications and content they find appealing therefore practitioners have a role in curating evidenced-based, suitable tech-based information (Bergin and Davies, 2020). Huby et al.’s (2017) tech study advocates the use of colour and games for those aged 5–10, and clear information for those aged 11–15, in order to increase accessibility and effectiveness. Huby et al. (2017) concludes children want clear, and reliable information without extended scrolling.

Technology offers positive psychosocial impact, through increasing connectivity and providing distraction (Nicholas and Chahauver, 2017; Maor and Mitchem, 2015; Lambert et al., 2014). Away from the web, Moerman and Jansens (2021) report use of robotic animals to comfort and distract children. Overall, research shows that technology increases patient engagement and could alleviate distress and anxiety in the absence of loved ones (Sawesi et al., 2016; Dawe et al., 2018).

## Discussion

There is increasing emphasis on capturing the narratives and centralising the voices of children, young people and other less-often heard groups (Bryan et al., 2019; Maynard et al., 2020; Sims-Schouten et al., 2022). Our scoping review has reviewed empirical studies which have done so. A scoping review approach enabled us to consider a wider range of evidence than a lone empirical study and mitigates some of the risks surrounding tokenism in co-production (Jasem et al., 2022).

Our review considers 22 studies through which to investigate our research questions.

Overwhelmingly the studies reflect emotional experiences for both children and parents (RQ1, RQ2) such as fear, stress, interpersonal relationships, play and information giving and gathering. Information (RQ3) reflects interpersonal communication over tech-based approaches in practice to date, with children’s own use of technology identified as a support mechanism. We were unable to find any studies which consider the needs of children and parents in combination. We have begun to address this here, as, although parent and child needs are distinct from one another, there is also clear symbiosis. Parents are able to care for their child more calmly and effectively when they themselves feel calm (Foster et al., 2016). This depends on skilful communication by healthcare teams including their ability to involve their child in the right way (subjectively speaking) (Huby et al., 2017).

Given sick children’s displacement from normal childhood experiences, opportunities for play and interaction are particularly important in hospitals, and such opportunities for children should be maximised (Jasem et al., 2022; Constantinou et al., 2021). The studies reveal a spectrum of experience for children and parents, from feeling unsafe, exposed and fearful, to happy, comforted and special, and play opportunities enable parents to feel supported and gain respite (Bryan et al., 2019; Kassa et al., 2017).

Quality of information giving and gathering has been identified as a key finding (Lernevall et al., 2021; Huby et al., 2017). The potential to involve technology here is evident, though we note the complexities in this area, as children may access irrelevant, inaccurate, or inappropriate information (Huby et al., 2017). This supports the notion that technology could support, but not replace, human interaction. Children select applications and content they find appealing therefore practitioners have a role in curating evidenced-based, suitable tech-based information. This could include co-production which has the potential to enable greater self-efficacy and ownership for decision making (Bergin and Davies, 2020; Sims-Schouten et al., 2022). Shilling et al. (2012), Kirk and Hinton (2019) and Wangmo et al. (2016) indicate that a combination of distraction through play, and reliable, age-appropriate information creates a sense of control so reducing anxiety. Thus, while technology boasts accessible self-help, further research is required as to the nuances of tech-based health interventions for children (Punukollu and Marques, 2019; Boydell et al., 2014).

The studies reveal children actively creating a pseudo-safe haven around themselves (Shilling et al., 2012; Plummer et al., 2021), indicating a deep sense of trauma. The play-centric studies (Bruins Slot et al., 2018; Lernevall et al., 2021; Dawe et al., 2018) illustrate child-friendly interventions can calm anxieties, although those for older children appear scant or non-existent and reflect them creating their own support, strategising with sick-child peers to process, take control and cope with altered bodies in a norm-obsessed world (Passos dos Santos et al., 2022; Heath et al., 2022).

### Limitations

Our review has brought together the experiences of children and parents in combination, which we understand to have been a previous gap. More work is required to understand this in greater depth, and to consider differences by gender, age, ethnicity and nationality as these factors are not currently represented. There is a clear indication of direct trauma for children and vicarious trauma for their parents. We suggest investigating trauma for families experiencing serious childhood illness could yield important findings for trauma-informed developments in healthcare provision and note a lack of focus on fathers. We did not consult with stakeholders beyond our funders at this stage. A further piece of work would prioritise co-production of tech-based and/or non-tech–based support with families, for families.

### Implications for practice

We have identified that age-appropriate, sensitive information giving is a priority for both children and parents. There are direct associations between good communication and the ability to cope, and poor communication, fear and mistrust. Play opportunities were sought in real-world and virtual spaces. We note that a whole-family perspective is adopted for children in hospital as the literature indicates positive hospital experiences for parent and child are mutually dependent on the support given to the other. In particular, parents consistently report that they are better able to care for their children and reassure them when they feel supported and well informed. Future developments for families should be co-constructed with them, retaining at heart the notion of a normal, developing child, displaced from normal things.

## Conclusion

Our research questions have been addressed and resulted in consistent findings which reflect very significant intersections found between parent and child needs and experiences. Answers to all three questions; 1. What do children experience during illness and treatment? 2. What do parents experience when their child is seriously ill in hospital? 3. What tech and non-tech interventions support children’s experience of in-patient care? Relate to emotional experience. While these are predominantly negative and frightening, they are alleviated by sensitive interpersonal and caring information giving. Emotional reassurance for children is supported by this information giving, alongside age-appropriate play and support for parents. Some gaps in provision for older children are identified. Close attention should be paid to normal childhood development, coexisting with serious childhood illness.

## References

[bibr1-13674935231168683] Bergin A Davies EB (2020) technology matters: mental health apps -- separating the wheat from the chaff. Child and Adolescent Mental Health 25(1): 51–53. DOI: 10.1111/camh.12363. 32063750 PMC7006755

[bibr2-13674935231168683] Boydell KM Hodgins M Pignatiello A , et al. (2014) Using technology to deliver mental health services to children and youth: a scoping review. J Can Acad Child Adolesc Psychiatry 23(2): 87–99. https://www.ncbi.nlm.nih.gov/pmc/articles/PMC4032077/. 24872824 PMC4032077

[bibr3-13674935231168683] Braun V Clarke V (2006) Using thematic analysis in psychology. Qualitative Research in Psychology 3(2): 77–101. DOI: 10.1191/1478088706qp063oa.

[bibr4-13674935231168683] Bruins Slot J Hendriks M Batenburg R (2018) Feeling happy and carefree: a qualitative study on the experiences of parents, medical clowns and healthcare professionals with medical clowns. International Journal of Qualitative Studies on Health and Well-Being 13(1): 1503909–1503910. DOI: 10.1080/17482631.2018.1503909. 30156995 PMC6116696

[bibr5-13674935231168683] Bryan G Bluebond-Langner M Kelly D , et al. (2019) Studying children’s experiences in interactions with clinicians: identifying methods fit for purpose. Qualitative Health Research. 29(3) 393–403 DOI: 10.1177/1049732318801358. 30270755

[bibr6-13674935231168683] Coad JE Shaw KL (2008) Is children’s choice in health care rhetoric or reality? a scoping review. Journal of Advanced Nursing 64(4): 318–327. DOI: 10.1111/j.1365-2648.2008.04801.x. 18990109

[bibr7-13674935231168683] Constantinou C Payne N van den Akker O , et al. (2021) A qualitative exploration of health-related quality of life and health behaviours in children with sickle cell disease and healthy siblings. Psychology Health 38: 1–22. DOI: 10.1080/08870446.2021.1955119. 34339316

[bibr8-13674935231168683] Crossen K (2017) Missed opportunities for adolescent friendly care in hospital. Journal of Paediatrics and Child Health 53: 1176–1179. DOI: 10.1111/jpc.13626. 28675486

[bibr9-13674935231168683] Darling SJ Hearps SJC Muscara F , et al. (2019) Psychological trajectories of mothers and fathers following their child’s diagnosis of a life-threatening illness or injury: a longitudinal investigation. Journal of Clinical Psychology 75(10): 1930–1942. DOI: 10.1002/jclp.22829. 31254362

[bibr10-13674935231168683] Davison G Kelly MA Thompson A , et al. (2020) Children’s and adolescents’ experiences of healthcare professionals: scoping review protocol. Systematic Reviews 9(9): 51. DOI: 10.1186/s13643-020-01298-6. 32145750 PMC7060982

[bibr11-13674935231168683] Davison G Kelly MA Conn R , et al. (2021) How do children and adolescents experience healthcare professionals? Scoping review and interpretive synthesis. BMJ Open 11:e054368. doi:10.1136/bmjopen-2021-054368. PMC8273482 34244289

[bibr12-13674935231168683] Dawe J Sutherland C Barco A , et al. (2019) Can social robots help children in healthcare contexts? a scoping review. BMJ Paediatrics Open 3: e000371. DOI: 10.1136/bmjpo-2018-000371. 30815587 PMC6361370

[bibr13-13674935231168683] Fancourt D Finn S (2019) What is the evidence on the role of the arts in improving health and well-being? A scoping review. Geneva, Switzerland: World Health Organization. Available at: https://apps.who.int/iris/handle/10665/329834. 32091683

[bibr14-13674935231168683] Foster K Young A Mitchell R , et al. (2017) Experiences and needs of parents of critically injured children during the acute hospital phase: a qualitative investigation. Injury 48(1): 114–120. DOI: 10.1016/j.injury.2016.09.034. 27692666

[bibr15-13674935231168683] Gjærde LK Hybschmann J Dybdal D , et al. (2021) Play interventions for paediatric patients in hospital: a scoping review. BMJ Open 11: e051957. DOI: 10.1136/bmjopen-2021-051957. PMC8314749 34312210

[bibr16-13674935231168683] Heath G Screti C Pattison H , et al. (2022) Understanding the impact of ‘wish-granting’ interventions on the health and well-being of children with life-threatening health conditions and their families: a systematic review. Journal of Child Health Care: For Professionals Working with Children in the Hospital and Community 26(3): 479–497. DOI: 10.1177/13674935211016712. 33966480

[bibr17-13674935231168683] Huby K Swallow V Smith T , et al. (2017) Children and young people’s views on access to a web-based application to support personal management of long-term conditions: a qualitative study. Child: Care, Health and Development 43(1): 126–132. DOI: 10.1111/cch.12394. 27554643

[bibr18-13674935231168683] Jasem ZA Lambrick D Randall DC , et al. (2022) The social and physical environmental factors associated with the play of children living with life threatening/limiting conditions: a Q methodology study. Child: Care, Health and Development 48(2): 336–346. DOI: 10.1111/cch.1293. 34806192

[bibr19-13674935231168683] Kassa AM Engvall G Engstrand Lilja H (2017) Young children with severe congenital malformations (VACTERL) expressed mixed feelings about their condition and worries about needles and anaesthesia. Acta Paediatrica (Oslo, Norway: 1992) 106(10): 1694–1701. DOI: 10.1111/apa.13973. 28672091

[bibr20-13674935231168683] Kirk S Hinton D (2019) I’m not what I used to be”: a qualitative study exploring how young people experience being diagnosed with a chronic illness. Child: Care, Health and Development 45(2): 216–226. DOI: 10.1111/cch.12638. 30652354

[bibr21-13674935231168683] Lambert V Coad J Hicks P , et al. (2014) Social spaces for young children in hospital. Child: Care, Health and Development 40(2): 195–204. DOI: 10.1111/cch.12638. 23294129

[bibr22-13674935231168683] Lernevall LST Moi AL Gjengedal E , et al. (2021) Parents’ lived experiences of parental needs for support at a burn centre. *International Journal of Qualitative Studies on Health and WellBeing* 16(1) 1–12 DOI: 10.1080/17482631.2020.1855749. PMC7808374 33427115

[bibr23-13674935231168683] Levac D Colquhoun H O’Brien KK (2010) Scoping studies: advancing the methodology. Implementation Science: IS 5(69): 69. https://implementationscience.biomedcentral.com/track/pdf/10.1186/1748-5908-5-69.pdf. 20854677 10.1186/1748-5908-5-69 PMC2954944

[bibr24-13674935231168683] Lewis P Klineberg E Towns S , et al. (2016) The effects of introducing peer support to young people with a chronic illness. Journal of Child and Family Studies 25(8): 2541–2553 DOI: 10.1007/s10826-016-0427-4.

[bibr25-13674935231168683] Lindström Nilsson M Funkquist E-L Edner A , et al. (2020) Children report positive experiences of animal-assisted therapy in paediatric hospital care. Acta Paediatrica (Oslo, Norway: 1992) 109: 1049–1056. DOI: 10.1111/apa.15047. 31597211

[bibr26-13674935231168683] Maalouf N Sidaoui A Elhajj IH , et al. (2018) Robotics in nursing: a scoping review. Journal of Nursing Scholarship: An Official Publication of Sigma Theta Tau International Honor Society of Nursing 50(6): 590–600. DOI: 10.1111/jnu.12424. 30260093

[bibr27-13674935231168683] Maor D Mitchem K (2015) Can technologies make a difference for hospitalized youth: findings from research. Journal of Computer Assisted Learning 31(6): 690–705. DOI: 10.1111/jcal.12112.

[bibr29-13674935231168683] Maynard E Barton S Rivett K , et al. (2020) Because ‘grown-ups don’t always get it right’: allyship with children in research – from research question to authorship. Qualitative Research in Psychology 18(4). DOI: 10.1090/14780887.2020.1794086.

[bibr30-13674935231168683] McKevitt C Topor M Panton A , et al. (2019) Seeking normality: parents’ experiences of childhood stroke. Child: Care, Health and Development 45(1): 89–95. https://pubmed.ncbi.nlm.nih.gov/30255632/#:∼:text=doi%3A%2010.1111/cch.12622. 30255632 10.1111/cch.12622

[bibr31-13674935231168683] Moerman CJ Jansens RM (2021) Using social robot PLEO to enhance the well-being of hospitalised children. Journal of Child Health Care: For Professionals Working with Children in the Hospital and Community 25(3): 412–426. https://pubmed.ncbi.nlm.nih.gov/32840383/#:∼:text=doi%3A%2010.1177/13674935209475. 32840383 10.1177/1367493520947503

[bibr32-13674935231168683] Nicholas DB Chahauver A (2017) Examining computer use by hospitalized children and youth. Journal of Technology in Human Services 35(4): 277–291. DOI: 10.1080/15228835.2017.1366886.

[bibr33-13674935231168683] Passos dos Santos R Macdonald ME Carnevale FA (2022) Moral experiences of children with medical complexity: a participatory hermeneutic ethnography in Brazil. Journal of Child Health Care 0(0): 136749352211121. DOI: 10.1177/13674935221112156. PMC10240624 36168769

[bibr34-13674935231168683] Pelentsov LJ Laws TA Esterman AJ (2015) The supportive care needs of parents caring for a child with a rare disease: a scoping review. Disability and Health Journal 8: 475–491. DOI: 10.1016/j.dhjo.2015.03.009. 25959710

[bibr35-13674935231168683] Plummer K McCarthy M McKenzie I , et al. (2021) Experiences of pain in hospitalized children during hematopoietic stem cell transplantation therapy. Qualitative Health Research, 31(12) 2247–2259 https://pubmed.ncbi.nlm.nih.gov/34369218/#:∼:text=doi%3A%2010.1177/10497323211034 161. 34369218 10.1177/10497323211034161

[bibr36-13674935231168683] Price J Hurley F Kiernan G .(2022) ‘Managing an unexpected life - a caregiver’s career’: parents’ experience of caring for their child with a non-malignant life-limiting condition. Journal of Child Health Care*.* DOI: 10.1177/13674935221132920. PMC11141083 36222549

[bibr37-13674935231168683] Punukollu M Marques M (2019) Use of mobile apps and technologies in child and adolescent mental health: a systematic review. Evidence-Based Mental Health 22: 161–166. DOI: 10.1136/ebmental-2019-300093. 31358537 PMC10270440

[bibr38-13674935231168683] Sawesi S Rashrash M Phalakornkule K , et al. (2016) The impact of information technology on patient engagement and health behavior change: a systematic review of the literature. JMIR Medical Informatics 4(1): e1. DOI: 10.2196/medinform.4514. 26795082 PMC4742621

[bibr39-13674935231168683] Sims-Schouten W Maynard E Pound M (2022) I hate having my mental health” – Making sense of mental health through coproduction and visual methods with young people with complex needs. Journal of Youth Studies: 1–19. DOI: 10.1080/13676261.2022.2101358.

[bibr40-13674935231168683] Shilling V Edwards V Rogers M , et al. (2012) The experience of disabled children as inpatients: a structured review and synthesis of qualitative studies reporting the views of children, parents and professionals. Child: Care, Health and Development 38(6): 778–788. DOI: 10.1111/j.1365-2214.2012.01372.x. 22372968

[bibr41-13674935231168683] Stewart M Letourneau N Masuda JR , et al. (2013) Online support for children with asthma and allergies. Journal of Family Nursing 19(2): 171–197. DOI: 10.1177/1074840713483573. 23559663

[bibr42-13674935231168683] Terras MM Ramsay J (2016) Family digital literacy practices and children’s mobile phone use. Frontiers in Psychology 7(1957) 1957–2011 https://www.frontiersin.org/articles/10.3389/fpsyg.2016.01957/full. 28066284 10.3389/fpsyg.2016.01957 PMC5179510

[bibr43-13674935231168683] Wangmo T Ruhe KM Badarau D , et al. (2016) Swiss paediatric oncology group. Parents’ and patients’ experiences with paediatric oncology care in Switzerland - satisfaction and some hurdles. Swiss Medical Weekly, 146 w14309. https://pubmed.ncbi.nlm.nih.gov/27124885/#:∼:text=10.4414/smw.2016.14309. 27124885 10.4414/smw.2016.14309

[bibr45-13674935231168683] Witt S Dellenmark-Blom M Flieder S , et al. (2019) Health -related quality of life experiences in children and adolescents born with esophageal atresia: a Swedish–German focus group study. Child: Care, Health and Development 45(1): 79–88. DOI: 10.1186/s13023-021-01748-x. 30221367

[bibr46-13674935231168683] Wright J Elwell L McDonagh JE , et al. (2015) ‘It’s hard but you’ve just gotta get on with it’ – The experiences of growing-up with a liver transplant. Psychology and Health 30(10): 1129–1145. DOI: 10.1080/08870446.2015.1024245. 25727924

